# Why does a leader’s other-oriented perfectionism lead employees to do bad things? Examining the role of moral disengagement and moral identity

**DOI:** 10.3389/fpsyg.2024.1290233

**Published:** 2024-01-29

**Authors:** Feng Jiang, Weipeng Zhang, Hongyan Zhang, Zhe Zhang

**Affiliations:** ^1^School of Business Administration, Jeonbuk National University, Jeonju, Republic of Korea; ^2^School of Sports Sciences, Tianjin Normal University, Tianjin, China; ^3^School of Business Administration, Shandong Women's University, Jinan, Shandong, China

**Keywords:** leadership, other-oriented perfectionism, moral disengagement, unethical pro-organizational behavior, moral identity

## Abstract

**Introduction:**

Moral disengagement is an essential concept in organizational behavioral ethics, as it is strongly related to employee behaviors and attitudes. What is not clear, however, is which leader traits are directly associated with employees’ moral disengagement and which are indirectly associated with unethical behavior. This study draws on a social cognitive perspective that links leaders’ other-oriented perfectionism (LOOP) with unethical employee behavior. Specifically, we propose that LOOP provides employees with excuses and encouragement to engage in unethical pro-organizational behavior (UPB).

**Methods:**

We analyzed data collected from 266 full-time employees at two-time points, and used mediated and moderated structural equation models to test the hypotheses, and the findings largely support our claims.

**Results:**

The results suggest that LOOP effectively promotes employees’ involvement in UPB. Moderated mediation tests suggest that the positive indirect impact of LOOP on employees’ unethical behavior via moral disengagement was attenuated by higher employees’ moral identity.

**Discussion:**

In summary, the results indicate that when leaders emphasize only perfection and make unrealistic demands on their employees, the latter perceive that engaging in unethical behavior is demanded by the leader, that the responsibility is not theirs, and thus they are more willing to engage in unethical behavior. This study discusses the implications of these findings from both practical and theoretical perspectives.

## 1 Introduction

As the economy continues to grow, people not only feel the changes brought about by the business environment but also witness many business scandals. Scandals like those committed by Enron, WorldCom, and Volkswagen (Lian et al., 2022) are often carried out by company employees or executives to benefit their organization or department. For example, during the Volkswagen emissions scandal in 2015, the company’s management board, led by then-CEO Martin Winterkorn, consistently rejected proposals to upgrade vehicle emissions controls because of costs (Ewing, 2016). Such behavior is “intended to promote the effective functioning of an organization or its members (e.g., leaders) and violate core societal values, mores, laws, or standards of conduct” (Umphress and Bingham, 2011, p. 622), is commonly referred to as unethical pro-organizational behavior (UPB; Umphress et al., 2010; Umphress and Bingham, 2011; Chen and Chen, 2021). Although these behaviors are in the organization’s interest, they can potentially have damaging consequences for the business in terms of other stakeholders (Xu and Lv, 2018), damaging the company’s reputation, and undermining public trust (Farasat and Azam, 2022). Nowadays, external stakeholders including the government, shareholders, communities, and customers, are pressuring management employees to diminish their unlawful and unethical behavior (Treviño et al., 2006). Therefore, we must explore the underlying motivations for employees’ UPB and examine the mechanisms that explain why employees participate in such unethical behaviors.

Leadership plays a prominent role in defining UPB (Umphress and Bingham, 2011) and is core to the formation of employees’ unethical behavior (Brown and Treviño, 2006; Graham et al., 2015). Factors such as supervisor Machiavellianism (Belschak et al., 2018), transformational leadership (Luan et al., 2022), bottom-line mentality (Kamran et al., 2023), and leader–member exchange (Tang et al., 2023) help explain employee participation in UPB and greatly improve our understanding of ethical behavior. However, there is little known about the potential role of leader perfectionism, which is central to effective leadership behavior (Otto et al., 2021), in predicting employees’ UPB. Perfectionism, the tendency to set unreasonably high-performance standards (Frost et al., 1990), is considered a valuable asset in the workplace because it increases efficiency (Ozbilir et al., 2015). Existing studies on workplace perfectionism have focused on self-oriented perfectionism, ignoring the role of other-oriented perfectionism (Kim, 2022). Other-oriented perfectionism holds unrealistic standards for others and judges them critically based on these standards (Shoss et al., 2015). They are more callous and may show some psychopathic attitudes and behaviors and are not above hurting others to achieve their personal goals (Stoeber, 2015). These characteristics can influence an individual’s performance in sacrificial dilemmas and ethical decision-making (Pletti et al., 2017). Leaders’ other-oriented perfectionism (LOOP) is particularly relevant in the workplace (Shoss et al., 2015), as organizations increasingly expect and demand almost impossible performance standards from employees (Ocampo et al., 2020; Cîrșmari et al., 2023). LOOP also promotes employee’s socially prescribed perfectionism (Smith et al., 2017, 2019). Socially prescribed perfectionism engages in unethical behavior for self-protection (Shagirbasha et al., 2023). Researchers have argued that leaders setting higher demands effectively improve performance (Guo et al., 2021) but have ignored the possibility that employees may use unethical practices to meet the leader’s standards.

To investigate the detrimental effects of other-oriented perfectionism, we introduced moral disengagement. Moral disengagement is a collection of cognitive legitimation mechanisms that allow individuals to engage in unethical behavior without pain, separate from intrinsic moral standards (Bandura et al., 1996). We argue that moral disengagement is a mediating mechanism between LOOP and employees’ UPB. Drawing upon social cognitive theory, we argue that since the traits of LOOP and the relative power that leaders have over their employees (Raven et al., 1998), employees may believe that their unethical behavior is justified to satisfy the leader’s perfectionism and therefore engage in UPB.

**Figure 1:**
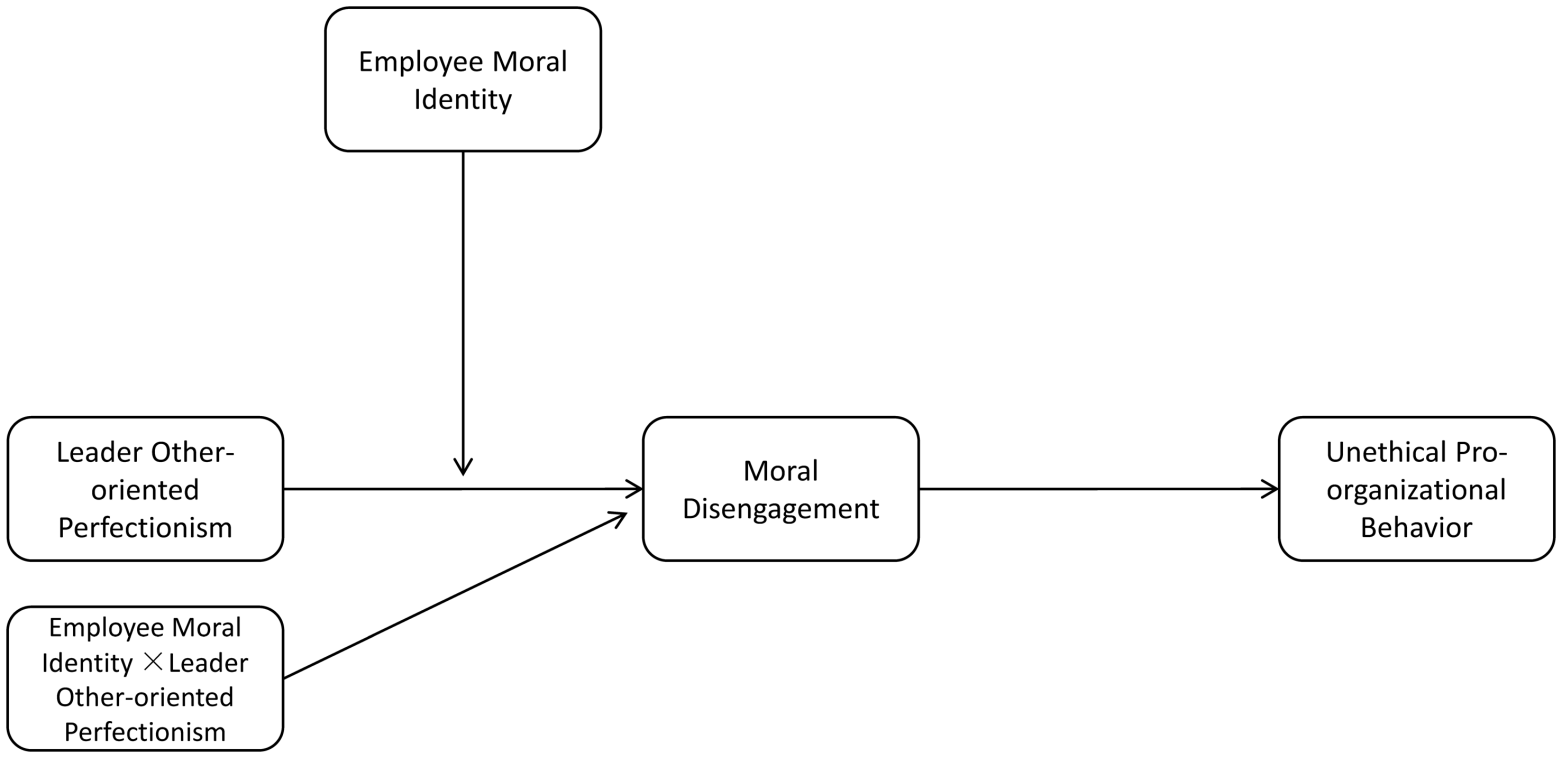
Conceptual model.

Furthermore, due to the lack of current research on the moderation mechanisms between perfectionism and work outcomes (Ocampo et al., 2020), we investigated how LOOP influences employees’ moral disengagement. To reduce the influence of LOOP on employees’ moral disengagement, this study proposes the level of moral identity as a theoretically relevant condition. Moral identity is a critical psychological mechanism for translating moral principles, judgments, or idealsku into action (Aquino et al., 2007). We suggest that employees respond differently to perceived LOOP depending on their moral identity level. When employees have a high level of moral identity, and even in the face of high demands from their leaders, they may adhere to their moral principles and be more inclined to adopt morally acceptable behaviors (see Figure 1).

The study makes several contributions to the existing literature. First, we investigate the antecedents of UPB to understand why other-oriented perfectionist leaders promote employees’ unethical behavior. This finding holds practical implications for perfectionist leadership development practices and for organizational interventions seeking to reduce the effects of ethical disengagement on unethical behavior. Second, this study echoes recent calls for research on workplace perfectionism (Ocampo et al., 2020) and shifts the central focus of the study from employees to leaders. As a result, we uncover the potential challenges affecting perfectionist leadership, which have very important practical implications for leadership development.

Third, by testing the moderating effect of moral identity, this study gains fresh insights into how individual differences in moral identity weaken (or strengthen) the effects of LOOP. Most research on UPB is cultural (Lian et al., 2022), and ethical intentions and behaviors are influenced by individual characteristics (Kish-Gephart et al., 2010), which helps explain why employees are more likely to demonstrate an intent to participate in UPB in similar organizational situations.

## 2 Literature review

### 2.1 Leader other-oriented perfectionism and unethical pro-organizational behavior

Perfectionism is a multidimensional personality structure (Flett et al., 2022). One of the most widely studied multidimensional models comprises the three dimensions distinguished by Hewitt and Flett (1991) from an interpersonal perspective: self-oriented, socially prescribed, and other-oriented perfectionism (Stoeber et al., 2020). Self-oriented perfectionists set high standards for their own behavior and value perfection (Davis et al., 2018). Socially prescribed perfectionists believe or perceive that significant others hold unrealistic standards, evaluating them critically, and pressuring them to be perfect (Hewitt and Flett, 1991; Hewitt et al., 2017). Other-oriented perfectionism directs the object of perfection toward others, has harsh standards for others, expects them to achieve perfection, and critically evaluates those who do not meet these expectations (Stoeber, 2018; Otto et al., 2021). Arbitrariness and unrealistic standards characterize other-oriented perfectionism generally (Hill et al., 1997). Since the workplace is a social environment in which employees frequently interact with colleagues and leaders (Shoss et al., 2015; Hussain et al., 2021), and many leaders are perfectionistic (Guo et al., 2020a) and expect and demand that their employees meet nearly impossible performance standards (Ocampo et al., 2020), it is a critical environment for studying the effects of other-oriented perfectionism.

The social cognitive theory suggests that contextual factors influence an individual’s cognition and behavior (Bandura, 1986). Leaders are important contextual factors that affect employees’ perceptions and behaviors in the workplace. LOOP sends a message of perfectionism to employees through the leader’s outward emotions, attitudes, and behaviors. LOOP reflects the leader’s standards and expectations of employees, and employees who cannot meet these standards and expectations are considered unqualified. Suppose employees are unable to meet the requirements of their leaders. In that case, they may suffer blame from their leaders (Xu et al., 2022) and lose opportunities for promotion and salary increases (Mesdaghinia et al., 2019). Therefore, employees may find it appropriate to strive to meet their leaders’ demanding standards to avoid blame and ignore other matters that may need to be prioritized (e.g., ethical guidelines; Welsh and Ordóñez, 2014). In such cases, employees pursue short-term performance results one-sidedly at the expense of long-term and larger outcomes (organizational and societal interests). Simultaneously, individuals’ ability, time, and willingness to reflect and think in a demanding leadership environment can be compromised and may narrow their scope of attention (Welsh and Ordóñez, 2014), elevating the unethical nature of the behavior.

In addition, LOOP results in unethical employee behavior, often shown in pro-organizational characteristics, due to the strong correlation between organizational and personal interests. Employees receive performance bonuses in the workplace that are inextricably linked to organizational performance. Employees can improve organizational performance through pro-organizational behavior and thus indirectly reap personal benefits (e.g., higher salaries and promotions). UPB is more insidious than direct, self-interested unethical behavior (e.g., embezzlement, theft).

Although no direct evidence supports the relationship between LOOP and employees’ UPB, high-performance expectations (Chen and Liang, 2017) and high goal setting (Ordonez and Welsh, 2015) influence employees’ engagement in UPB. Difficult and unmet goals have been found to motivate employees to behave unethically (Fukushima and Yamada, 2023). Furthermore, Chen and Chen (2021) indicated that employees under performance pressure would participate in UPB. In summary, the study proposes the following hypotheses:

*H1*: LOOP may positively impact employee UPB.

### 2.2 Mediating effect of moral disengagement

Through what mechanism does LOOP impact an employee’s UPB? According to the social cognitive theory, environmental factors influence behavior through the psychological mechanisms of the self-system (Bandura, 2001). The social cognitive theory establishes moral subjects in a self-regulatory system that operates through self-monitoring, judgment, and self-reactivity (Bandura et al., 1996). As part of this system, individuals construct moral standards as guides for action and deterrents; they develop guilt and self-condemnation when acting against their moral standards (Bandura, 1991, 1999). However, this self-regulatory system can only function if activated. Many social and psychological strategies cause self-regulatory systems to fail (Bandura, 2002), leading individuals to act immorally with no guilt or shame (Bandura, 1999; Tsang, 2002). Bandura (1999) refers to the psychological mechanism that causes the breakdown of this self-regulatory system as moral disengagement, which includes three broad dimensions. First, individuals may distort the outcomes of unethical behaviors through cognitive reconstructions (moral justifications, advantageous comparisons, and euphemistic labeling), making them appear less damaging or unethical (Bandura et al., 1996; Bandura, 1999). Second, individuals might weaken their role in unethical behavior by displacing and diffusing responsibility. For example, employees may perceive unethical behavior as acceptable by transferring responsibility to authority figures (Fehr et al., 2020). Finally, individuals may deny the harm experienced by the victim through the distortion of consequences, attribution of blame mechanisms, and dehumanization.

Some scholars argue that subordinates may activate moral disengagement mechanisms under high goals and high-performance pressure (Ogunfowora et al., 2022; Khan et al., 2023). Building on these studies, we suggest that LOOP creates the right conditions for employees to initiate moral disengagement mechanisms. Failure to meet perfectionist leaders’ high standards and demands may disappoint leaders and result in negative work outcomes (Welsh et al., 2020). Employees who meet high demands are rewarded and praised for doing so. The benefits of meeting high expectations may also justify employees’ unethical behavior. For example, an employee’s unethical behavior to meet the high-demand of the leader may be interpreted as serving the leader because of the leader’s high demands, transferring responsibility to the leader who demands perfectionism to avoid moral condemnation, thus activating moral disengagement. In addition, since the targets of UPB are often groups outside the firm, employees are prone to blaming mistakes on the victim’s misconduct or the cruelty of market competition (Chen and Liang, 2017).

Since moral disengagement invalidates the cognitive connection between unethical conduct and the self-approval process that prevents such behavior, morally disengaged individuals are unlikely to be aware of a moral problem and more likely to engage in unethical behavior (Schwartz, 2016). In other words, when individuals experience moral disengagement, they are convinced that ethical norms are not applicable to their current context. This is because they are unaware of the moral consequences of their actions and are overly concerned with the pro-organizational aspects of unethical behavior (Yan et al., 2021). Ethical disengagement has been shown to have a positive effect relationship on subsequent unethical behavior in various organizations and industries (Moore et al., 2012; Ebrahimi and Yurtkoru, 2017; Shaw et al., 2020). Thus, employees with a tendency toward moral disengagement are more likely to engage in UPB. Therefore, we argue that LOOP activates employees’ moral disengagement mechanisms to promote UPB at work.

*H2*: Moral disengagement mediates the influence of LOOP on employee UPB.

### 2.3 Moderating role of moral identity

Although LOOP is likely to activate moral disengagement mechanisms in employees, it is essential to recognize the effect of individual differences in this relationship. We believe that not all employees under a perfectionist leader engage in moral disengagement. In this context, our study places particular emphasis on moral identity as a variable to describes individual differences as “a self-conception formed around a series of moral traits, including traits like honesty, compassion, or loyalty” (Aquino and Reed II, 2002). This reflects the significance of being ethical in an individual’s identity (Hardy and Carlo, 2011), and is considered to be an important bridge to connect the moral gap between moral cognition and behavior (Blasi, 1983; Guo et al., 2019).

Individuals with a strong moral identity regard moral values are essential to shaping their identity (Wang et al., 2017), place greater value on moral cognition and behavior, and are more likely to have a higher moral awareness of the moral implications of a situation than those who have weaker moral identity (DeCelles et al., 2012). Moreover, people with a strong moral identity feel a stronger moral obligation to attend to the needs and concerns of the outgroup (Aquino et al., 2007; Winterich et al., 2009) and to be more generous in their attitudes toward the outgroup (Reed II and Aquino, 2003; Lefebvre and Krettenauer, 2019). When faced with the high demands of a perfectionist leader, employees with a high moral identity make behavioral choices that consider the interests of groups outside the company and the harm their actions may cause to others, thus effectively preventing moral disengagement. In comparison, employees with a weak moral identity place less emphasis on the discipline of moral traits and are less likely to consider the interests of the outgroup. When employees accomplish tasks from other-oriented perfectionist leaders, they are more likely to explain their behavior as compelled by the authority and commands of their leaders to engage in moral disengagement. We propose a third hypothesis following this reasoning:

*H3*: Employees’ moral identity negatively moderates the relationship between LOOP and moral disengagement, in such a way that this relationship is weaker with higher levels of moral identity than with lower levels.

Building on these analyses and hypotheses, we propose a moderated mediation model that the effect of LOOP on UPB through moral disengagement is likely affected by moral identity. Namely, employees who have strong moral identity might adhere to their moral perceptions and ethical behaviors and exhibit a reduced propensity for engaging in UPB when faced with high demands from perfectionist leaders. Employees who have weak moral identity are more prone to participate in UPB through moral disengagement mechanisms (e.g., dehumanization or responsibility transfer) when faced with high demands from perfectionist leaders. Therefore, we propose the following hypotheses:

*H4*: The indirect positive effects of LOOP on employees’ UPB via moral disengagement are weaker for those with high moral identity.

## 3 Materials and methods

### 3.1 Sampling criteria and procedure

To test our hypotheses, we designed and conducted a multi-organizational, two-point time survey. We drew on a sample of Chinese firms in the manufacturing, education, information technology, healthcare, and retail sectors to improve the generalizability of our paper. We gathered data in two phases separated by a 4-week interval to avoid potential problems associated with common method bias (Podsakoff et al., 2003). A 4-week interval enables changes in employee attitudes and behaviors while maintaining a stable work environment for employees (Daniels and Guppy, 1994). In the first time frame (Time 1), a questionnaire containing demographics was sent to 360 participants who were also asked to rate their LOOP, moral identity, and moral disengagement. We received 300 responses, with an 83.33% response rate. In the second time frame (Time 2), we distributed questionnaires to the 300 participants who completed the Time 1 questionnaire, asking them to rate UPB and social desirability, and received responses from 266 participants with an 88.67% response rate. Thus, the final sample comprised 266 employees.

Among the 266 employees, 116 (43.6%) were female compared to 150 (56.4%) males, the mean age was 28.66 years (SD = 4.92), and the mean tenure was 3.52 years (SD = 4.08). Regarding educational level, 15.80% had completed junior college and below, 52.60% held an undergraduate degree, and 31.60% had a master’s degree and above. General employees accounted for the majority (68.40%), followed by first-line (15.40%), middle (13.90%), and senior managers (2.30%). The following industries were represented in the sample: healthcare was 10.50%; information technology, 17.30%; retail, 25.60%; education, 14.70%; manufacturing, 18.00%; and other, 13.9%.

### 3.2 Measurements

We utilized a 7-point Likert scale (ranging from 1 = strongly disagree to 7 = strongly agree) to evaluate all measurement instruments, with the exception of the control variables. Given that the original measures used were in English, three researchers fluent in English and Chinese used a standard translation and back-translation (Brislin, 1970) process to accurately translate the questionnaire questions into Chinese. This process was implemented to ensure the semantic accuracy and content validity of the Chinese translations of the measures.

#### 3.2.1 Leader other-oriented perfectionism (time 1)

We used a brief version of the five items developed by Hewitt and Flett (1991) and reduced them as per Hewitt et al. (2008) to allow employees to rate their LOOP. To capture employees’ perceptions of their LOOP, rather than the leader’s perceptions, we replaced the word “I” with “my leader.” Sample items include “My leader has high expectations for the people who are important to him/her” and “My leader cannot be bothered with people who will not strive to better themselves” (Cronbach’s alpha = 0.89).

#### 3.2.2 Moral disengagement (time 1)

Moore et al. (2012) eight-item scale was used to measure employees’ moral disengagement. An example item is: “Taking personal credit for ideas that were not your own is no big deal” (Cronbach’s alpha = 0. 92).

#### 3.2.3 Moral identity (time 1)

Consistent with prior studies (Chowdhury and Fernando, 2014; Wang et al., 2021), we used the internalization subscale of moral identity developed by Aquino and Reed II (2002) to measure employees’ moral identity. This dimension has a stronger predictive effect on individuals’ moral concerns and behaviors than the moral symbolization dimension of moral identity (Shao et al., 2008). This scale describes nine ethical characteristics (caring, compassionate, fair, friendly, generous, helpful, hard-working, honest, and kind). We asked participants to envision how an individual embodying these ethical traits would think, feel, and act, and then asked them to rate the five items. An example item is: “It would make me feel good to be a person with these characteristics” (Cronbach’s alpha = 0.90).

#### 3.2.4 Unethical pro-organizational behavior (time 2)

Umphress et al. (2010) six-item scale was used to measure UPB. An example item is: “If it would help my organization, I would misrepresent the truth to make my organization look good” (Cronbach’s alpha = 0.89).

#### 3.2.5 Control variables

Our analysis used six employee demographics as control variables, including gender, age, education, tenure, job position, and industry, and these variables were found to correlate with employees’ moral disengagement, moral identity, and unethical behavior (Betz et al., 1989; Zhang et al., 2018). For instance, age is correlated with employees’ UPB (Yan et al., 2021; Luan et al., 2022), and gender significantly affects employees’ UPB (Guo et al., 2020b). To measure participants’ social desirability in responding to the self-report questionnaire, we adopted a 6-item scale from a reduced version of the Marlowe-Crowne Social Desirability Scale developed by Reynolds (1982). Items included “I sometimes feel resentful when I do not get my way,” “There have been occasions when I took advantage of someone,” “I’m always willing to admit it when I make a mistake,” “I sometimes try to get even rather than forgive and forget,” “There have been times when I was quite disgusted by the good fortune of others,” and “I am sometimes irritated by people who ask for favors of me.” Participants responded on a 2-point scale (1 = yes, 2 = no), with higher scores indicating higher social desirability of the response (Cronbach’s alpha = 0.86).

## 4 Results

### 4.1 Descriptive statistics

We performed descriptive statistics and correlation analyses of the study variables using SPSS 26.0. Table 1 shows the means and standard deviations of the variables and the correlation coefficients between the variables. All the measures with an acceptable level of reliability. LOOP is positively correlated with moral disengagement (*r* = 0.29, *p* < 0.01), UPB (*r* = 0.32, *p* < 0.01), and not significantly correlated with moral identity (*r* = 0.08, *p* > 0.05). Moral disengagement was negatively correlated with moral identity (*r* = −0.54, *p* < 0.01) and positively correlated with UPB (*r* = 0.44, *p* < 0.01). Moral identity is negatively correlated with UPB (*r* = −0.14, *p* < 0.05). As age was strongly correlated with tenure and position, we excluded age from subsequent analysis.

**Table 1:** Descriptive statistics, correlations, and reliabilities.

Variables	*M*	*SD*	1	2	3	4	5	6	7	8	9	10
1.Gender	0.44	0.50										
2.Age	28.66	4.92	−0.16^**^									
3.Tenure	3.52	4.08	0.01	0.76^**^								
4.Education	2.16	0.67	−0.15^*^	−0.10	−0.26^**^							
5.Position	1.50	0.82	−0.03	0.61^**^	0.57^**^	−0.19^**^						
6.Social desirability	1.52	0.35	0.04	−0.03	−0.03	−0.09	−0.05	(0.86)				
7.LOOP	5.08	1.32	0.01	0.03	0.09	0.10	−0.05	−0.09	(0.89)			
8.Moral disengagement	3.56	1.34	−0.02	−0.06	−0.02	0.11	−0.01	−0.13^*^	0.29^**^	(0.92)		
9.Moral identity	4.98	1.58	−0.03	−0.03	−0.04	−0.04	−0.03	0.08	0.08	−0.54^**^	(0.90)	
10.UPB	3.87	1.23	−0.22^**^	−0.06	−0.07	0.17^**^	−0.05	−0.22^**^	0.32^**^	0.44^**^	−0.14^*^	(0.89)

*N* = 255. SD is the standard deviation. Internal reliabilities (alpha coefficients) of the constructs are given in parentheses on the diagonal. Gender = “male” (1), “female” (2); education = junior college and below (1), undergraduate (2), master’s and above (3).*p < 0.05.**p < 0.01.

Before examining the hypotheses, we tested the fit of the conceptual model using confirmatory factor analysis (CFA) in Mplus 8.3 (Muthén and Muthén, 2017). A five-factor model (LOOP, moral identity, moral disengagement, social desirability, and UPB) was specified and demonstrated a good fit with the data: χ^2^(395) = 635.48, CFI = 0.948, and TLI = 0.943. The CFA model also has a better fit than the alternative measurement models (see Table 2).

**Table 2:** Confirmatory factor analysis of measurement models.

Model	*χ^2^*	*df*	CFI	TLI	RMSEA	SRMR	Δ *χ^2^*/ Δ *df*
Hypothesized five-factor model (LOOP, MI, MD, SD, UPB)	635.48	395	0.95	0.94	0.05	0.05	_
Four-factor model (LOOP, MI, SD, MD + UPB)	1247.94	399	0.82	0.80	0.09	0.09	612.46(4) ^***^
Three-factor model (LOOP+MI, SD, MD + UPB)	2088.52	402	0.64	0.61	0.13	0.17	840.58(3) ^***^
Two-factor model (LOOP+MI + SD, MD + UPB)	2772.51	404	0.49	0.45	0.15	0.19	683.99(2) ^***^
One-factor model (LOOP+MI + SD + MD + UPB)	3056.79	405	0.43	0.39	0.16	0.16	284.28(3) ^***^

+ indicates the combination of two factors as a single factor. LOOP, leaders’ other-oriented perfectionism; MI, moral identity; SD, social desirability; MD, moral disengagement; UPB, unethical pro-organizational behavior.

Our study ensured participant anonymity at the time of the ex-ante survey, strictly followed validated instruments for measuring the variables, collected data from different time periods, and temporarily separated the causality of the variables to reduce the threat of common method variance (CMV; Darren et al., 2022). Ex-post, we used Harman’s single-factor test (Podsakoff et al., 2003) to check for CMV arising from data from a single source. The results showed the presence of more than one factor, with the first component explaining only 28.12% of the overall variance. Although neither ex-ante nor ex-post tests can conclusively rule out the presence of CMV (Richardson et al., 2009), these results suggest that CMV was not a major problem in this study.

### 4.2 Structural model for testing hypotheses

We tested hypotheses 1–4 by performing a path analysis using Mplus 8.3. The results after controlling for gender, tenure, education level, position, and social desirability are in Table 3. H1, that LOOP is positively associated with employees’ UPB, is supported (*β* = 0.36, SE = 0.06, *p* < 0.001; see Table 3: Model 4).

**Table 3:** Mediating role of moral disengagement.

Dependent variables	Moral disengagement						Unethical pro-organizational behavior			
	Model 1		Model 2		Model 3		Model 4		Model 5	
Measure	β (SE)	*p*	β (SE)	*p*	β (SE)	*p*	β (SE)	*p*	β (SE)	*p*
Gender	0.01(0.06)	0.92	0.01(0.06)	0.99	−0.01(0.05)	0.82	−0.19(0.06)	0.00	−0.19(0.05)	0.00
Tenure	0.01(0.08)	0.95	−0.06(0.08)	0.43	−0.11(0.06)	0.08	−0.10(0.07)	0.15	−0.08(0.07)	0.22
Education	0.10(0.07)	0.12	0.06(0.06)	0.32	0.02(0.05)	0.67	0.08(0.06)	0.20	0.06(0.06)	0.33
Position	0.01(0.08)	0.98	0.05(0.07)	0.51	0.05(0.06)	0.42	0.04(0.07)	0.63	0.02(0.07)	0.79
Social desirability	−0.13(0.07)	0.04	−0.11(0.06)	0.09	−0.03(0.05)	0.49	−0.19(0.06)	0.00	−0.15(0.06)	0.01
LOOP			0.32(0.06)	0.00	0.39(0.05)	0.00	0.36(0.06)	0.00	0.24(0.06)	0.00
Moral disengagement									0.38(0.06)	0.00
Moral identity					−0.64(0.04)	0.00				
*Interaction					−0.13(0.05)	0.02				
*R^2^*	0.03		0.13		0.54		0.23		0.35	
*ΔR^2^*	0.01		0.11		0.53		0.21		0.33	
*F*	1.61		6.45^***^		37.71^***^		12.89^***^		19.85^***^	
Index of moderated mediation				Index (SE)		LLCI		ULCI		
Moral identity				−0.05		−0.10		−0.01		

*N* = 266. β is the standardized regression coefficient, and SE is the standard error. Bootstrap sample size = 5,000. Gender = male (0); female (1); education = junior college and below (1), undergraduate (2), master’s and above (3); LOOP: leaders’ other-oriented perfectionism, *Interaction = leaders’ other-oriented perfectionism × coping self-efficacy, LLCI: lower limit confidence interval, ULCI: upper limit confidence interval; ^***^p < 0.001.

As predicted, the results for Model 2 and Model 5 demonstrated that the positive indirect influence of LOOP on UPB through moral disengagement was statistically significant (*β* = 0.12, *p* < 0.001, 95% C.I. [0.07, 0.20]), proposing a complementary mediation (Zhao et al., 2010). Therefore, H2 is supported.

**Figure 2:**
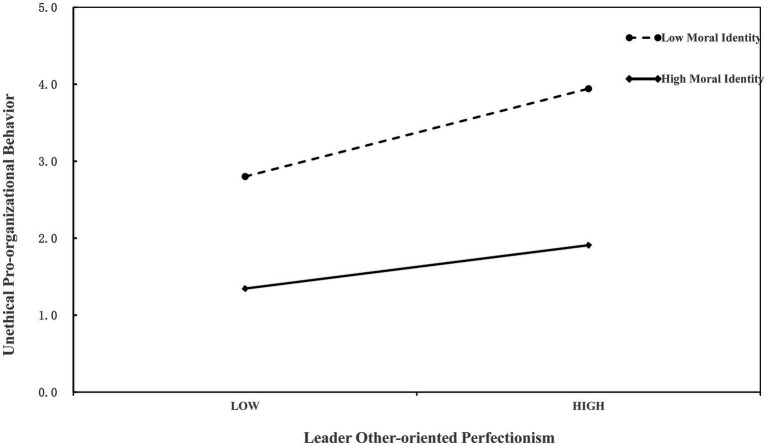
Interaction of moral identity and LOOP on moral disengagement.

To test H3, that the interaction between LOOP and employee moral identity significantly affected moral disengagement (*β* = −0.13, *p* < 0.05; see Table 3: Model 3) we graphed simple slopes for the relationship between LOOP and moral disengagement at low (− 1 SD) and high (+1 SD) levels of moral identity (Figure 2). Simple slope analyses indicated that the relationship between LOOP and moral disengagement was weaker when moral identity was high (*β* = 0.30, *p* < 0.001, 95% C.I. [0.13, 0.46]) and stronger when moral identity was low (*β* = 0.58, *p* < 0.001, 95% C.I. [0.38, 0.77]). Thus, H3 is supported.

H4 predicted that moral identity moderates the mediating effect of moral disengagement. As shown in Table 3, the moderated mediation effect index for UPB was significant (index = −0.05, 95% C.I. [−0.10, −0.01]), which suggests that a moderated mediation effect did exist. Specifically, Our results showed that the indirect effect of LOOP via moral disengagement on UPB became weaker and significant at higher levels of moral identity (estimate = 0.11, 95% C.I. [0.04, 0.18]). The indirect effect of LOOP via moral disengagement on UPB became stronger and significant when moral identity was low (estimate = 0.22, 95% C.I. [0.12, 0.31]). Thus, H4 is supported.

## 5 Discussion

Using data collected over two periods, we found that LOOP was associated with employees’ UPB and mediated by moral disengagement, whereas moral identity weakened this relationship. Over the next few sections, we expound upon the theoretical and practical implications of these results, address limitations, and delineate directions for future research.

### 5.1 Theoretical contributions

Our study contributes to the literature in four aspects. First, it deepens our comprehension of the impact of workplace LOOP on employee perceptions and behaviors. Previous research related to perfectionism has focused on clinical and educational contexts (Shafran et al., 2002; Lee and Anderman, 2020), scholars have only recently emphasized the significance of perfectionism in the workplace (Kleszewski and Otto, 2020; Ocampo et al., 2020). Our study investigated the adverse outcomes of LOOP in the workplace. Most studies on workplace leadership perfectionism have focused on leaders’ self-critical nature (Guo et al., 2020a; Song et al., 2022). By contrast, our study considers an interpersonal perspective, focusing on the impact of leaders’ perfectionist demands and criticisms of others (employees).

Second, it contributes to the field of UPB research. We complement previous research by highlighting the impact of high standards and demands from LOOP on employees’ UPB. Although some prior research has explored the influence of factors such as leadership style, work context, etc. (Mishra et al., 2021) on UPB, there is still a lack of research considering UPB from the perspective of leaders’ personality traits. We extend the antecedents of employees’ UPB to leaders’ perfectionist characteristics.

Third, our paper examined the mediating role of moral disengagement on LOOP and employees’ UPB for the first time. As in Bandura (1999) theory of social cognitive theory, the findings of our paper suggest that moral disengagement can be used as a cognitive mechanism to explain the association between LOOP and employees’ UPB. This means that leaders’ high expectations and standards for employees provide employees with excuses for moral disengagement, and employees may shift their blame to leaders, increasing their involvement in UPB.

Fourth, we examined the moderating mechanisms between LOOP and employee moral disengagement, echoing Ocampo et al. (2020) call for more concern with boundary conditions when studying the effects of workplace perfectionism and work outcomes. This study addresses this gap by demonstrating that moral identity plays a key moderating part in weakening the adverse effects of workplace perfectionism. That is, employees who have high moral identity adhere to their moral perceptions while fulfilling the high demands of their leaders, reducing moral disengagement, and discouraging employees from engaging in UPB. By finding that moral identity is an important boundary condition, we developed and extended theories related to the conditions under which LOOP encourages employees to engage in UPB.

### 5.2 Practical implications

Our findings on the moral consequences of LOOP have important implications for managers and leaders. First, many leaders have perfectionist tendencies (Guo et al., 2020a), which are becoming increasingly common in the workplace (Ocampo et al., 2020). Our findings suggest that to reduce employees’ unethical behavior, leaders must understand the potential negative consequences of the other-oriented perfectionist personality, especially on employees, and develop a more balanced approach to achieving high standards and expectations. Specifically, we encourage leadership development workshops to raise awareness of the potentially harmful outcomes of LOOP practices. Additionally, managers should explicitly prohibit UPB while emphasizing high standards and requirements.

Second, the significant mediating effect of employee moral disengagement calls for managers’ awareness of the critical importance of mitigating employees’ tendency for moral disengagement and curbing the culture of moral disengagement in organizations. Moral disengagement is a moldable social cognitive orientation influenced by external social contexts (Hystad et al., 2014). We suggest that employees with low levels of moral disengagement should be explicitly sought after during the recruitment process through questionnaires and scenarios (He et al., 2019), as these employees will have difficulty finding valid reasons and excuses for their unethical behavior. This can also be achieved by creating an ethics climate that communicates values clearly to employees, for example, by establishing clear cultural norms about ethical and unethical employee behaviors or by fostering a collective climate valuing ethics. In addition, leaders and managers should focus on the means to achieve their goals. When managers value these means, shifting their ethical responsibilities to their leaders may be difficult.

Third, this study highlights the important role of individual differences in employees’ moral identity in weakening the negative influence of perfectionism and reducing their moral disengagement and unethical behavior. Wang et al. (2021) showed that moral identity is plastic and based on developmental experiences related to specific social roles, like work roles. Therefore, organizations can endeavor to expedite the cultivation of moral identity grounded in job roles through developmental interventions and establish ethical standards to select, train, and reward employees. In addition, managers can clearly communicate to job applicants their recognition of ethical values and s recruit employees with high levels of moral identity (Wang et al., 2019).

Finally, this study explains how workplace perfectionism leads to UPB through moral disengagement based on social cognitive theory. Moral identity is considered an important bridge to bridge the ‘moral gap’ between moral cognition and moral conduct (Blasi, 1983). We focused primarily on the moderating effect of moral identity beyond the main effect of moral identity on ethical conduct, which is consistent with previous research (Wang et al., 2017). To better connect cognitive and behavioral frameworks, according to Kristjánsson (2010) and Darnell et al. (2019), we emphasize that employees’ moral education is an ongoing effort. Organizations can help employees internalize the ability to respond correctly to various situations by training them in critical judgment, role modeling, and guided activities (Kristjánsson, 2010).

### 5.3 Limitations and future research

As with other empirical research, this study has some limitations. First, it examined only one particular boundary condition, moral identity, and how it affected the relationship between LOOP and UPB. We speculate that other exciting and significant boundary conditions may also exist. For example, employees’ perceived motivations for LOOP may vary, as may their reactions. When employees perceive that the high demands of LOOP are motivated only by the leader’s selfish desires and are exploitative, they are more likely to participate in moral disengagement. Conversely, employees are less likely to initiate ethical disengagement in this scenario because the leader is concerned that their below-standard performance may make them look bad or because the leader wants to help them succeed (Shoss et al., 2015)—that is, when they have pro-social motivations. Future research could examine the effects of different motivations of leaders (self-interested motives/prosocial motives) on the relationship between LOOP and employees’ moral cognition, and behavior.

Second, we controlled only for social desirability and not for other variables, such as the dark triad (psychopathy, Machiavellianism, and narcissism). Stoeber (2014) found that other-oriented perfectionism had a unique positive relationship with the dark triad. Future studies could further examine our findings by controlling for the dark triad in the study design.

Third, due to the possible limitations of the research method or sample, there is no correlation between LOOP and employee moral identity in our findings. Prior research has shown that organizations as a context can not only promote certain ethical patterns, they can also influence individual moral identity (Huhtala et al., 2019). LOOP, as a context, creates external pressures on others in the social network (Smith et al., 2017), and to get the job done, employees may succumb to external pressures, even to the detriment of their personal values (Huhtala et al., 2019). Future research could improve measurement tools or research methods (e.g., multilevel designs) to explore whether this characteristic of leaders puts pressure on employees and affects their moral identity and ethical behavior. This is critical to advancing the theoretical understanding and practical implications of organizational ethics.

Finally, although the problem of CMV was somewhat reduced by collecting data over two periods, we collected data from the same source and used data from only two time points. This may have overlooked dynamic changes that may have arisen at T3. This study aimed to explore the impact of employees’ perceived LOOP on unethical behavior, which appears to be more appropriate for all structures that use self-report measures. Although relevant measures were taken to reduce and examine this problem, future research could add time points or use multiple sources and experimental designs.

## 6 Conclusion

In this study, we investigated the possible negative effects of LOOP on employees’ moral disengagement and UPB. Understanding the negative effects of perfectionism can be leveraged to help organizations obtain the benefits associated with perfectionism without incurring unexpected costs. We hope that our research will inspire scholars to further explore the various impacts of perfectionism.

## Data availability statement

The raw data supporting the conclusions of this article will be made available by the authors, without undue reservation.

## Ethics statement

The studies involving humans were approved by Ethics Committee of Tianjin Normal University (Ethics approval number: 2023071201). The studies were conducted in accordance with the local legislation and institutional requirements. Written informed consent to participate in this study was not required from the participants in accordance with the national legislation and the institutional requirements.

## Author contributions

FJ: Conceptualization, Data curation, Investigation, Methodology, Writing – original draft, Writing – review & editing. WZ: Conceptualization, Data curation, Investigation, Methodology, Writing – original draft, Writing – review & editing. HZ: Conceptualization, Data curation, Investigation, Methodology, Writing – original draft, Writing – review & editing. ZZ: Conceptualization, Data curation, Investigation, Writing – original draft, Writing – review & editing.
